# Genetic variation in populations of the earthworm, *Lumbricus rubellus*, across contaminated mine sites

**DOI:** 10.1186/s12863-017-0557-8

**Published:** 2017-11-17

**Authors:** Craig Anderson, Luis Cunha, Pierfrancesco Sechi, Peter Kille, David Spurgeon

**Affiliations:** 1 Biological and Environmental Sciences, School of Natural Sciences, University of Stirling, Stirling, FK9 4LA UK; 2 Centre for Ecology and Hydrology, Maclean Building, Benson Lane, Wallingford, OX10 8BB UK; 3 School of Biosciences, University of Cardiff, Main Building, Museum Avenue, Cardiff, CF10 3AT UK; 4 Embrapa Florestas, Estrada da Ribeira km. 111, Colombo, PR 83411-000 Brazil

**Keywords:** Earthworms, RADseq, Ecotoxicology, Population genomics, Adaptation, Arsenic, Lead

## Abstract

**Background:**

Populations of the earthworm, *Lumbricus rubellus*, are commonly found across highly contaminated former mine sites and are considered to have under-gone selection for mitigating metal toxicity. Comparison of adapted populations with those found on less contaminated soils can provide insights into ecological processes that demonstrate the long-term effects of soil contamination. Contemporary sequencing methods allow for portrayal of demographic inferences and highlight genetic variation indicative of selection at specific genes. Furthermore, the occurrence of *L. rubellus* lineages across the UK allows for inferences of mechanisms associated with drivers of speciation and local adaptation.

**Results:**

Using RADseq, we were able to define population structure between the two lineages through the use of draft genomes for each, demonstrating an absence of admixture between lineages and that populations over extensive geographic distances form discrete populations. Between the two British lineages, we were able to provide evidence for selection near to genes associated with epigenetic and morphological functions, as well as near a gene encoding a pheromone. Earthworms inhabiting highly contaminated soils bare close genomic resemblance to those from proximal control soils. We were able to define a number of SNPs that largely segregate populations and are indicative of genes that are likely under selection for managing metal toxicity. This includes calcium and phosphate-handling mechanisms linked to lead and arsenic contaminants, respectively, while we also observed evidence for glutathione-related mechanisms, including metallothionein, across multiple populations. Population genomic end points demonstrate no consistent reduction in nucleotide diversity, or increase in inbreeding coefficient, relative to history of exposure.

**Conclusions:**

Though we can clearly define lineage membership using genomic markers, as well as population structure between geographic localities, it is difficult to resolve markers that segregate entirely between populations in response to soil metal concentrations. This may represent a highly variable series of traits in response to the heterogenous nature of the soil environment, but ultimately demonstrates the maintenance of lineage-specific genetic variation among local populations. *L. rubellus* appears to provide an exemplary system for exploring drivers for speciation, with a continuum of lineages coexisting across continental Europe, while distinct lineages exist in isolation throughout the UK.

**Electronic supplementary material:**

The online version of this article (10.1186/s12863-017-0557-8) contains supplementary material, which is available to authorized users.

## Background

Monitoring life-history parameters among organisms is routinely used as a means of establishing the risks imposed by pollutants on natural populations [1, 2]. In knowing how classical endpoints vary, e.g. growth, reproduction and survival, researchers can determine the short-term effects of exposure to lab-based cohorts and are able to make inferences as to the effects on natural populations [3–5]. While these are useful for recognising the effects of exposure within a single generation, effects over multiple generations, such as changes in genetic diversity associated with selection, can be more subtle. What’s more, increased inbreeding depression can suggest a loss of adaptive capacity in affected populations, and can provide ecotoxicologists with valuable insights into demographic changes underlying population genetic end points [6, 7].

Identification of regions of the genome under selection is empowered with the availability of high-throughput sequencing methods such as Restriction-site Associated DNA sequencing (RADseq), which allows for identification of markers across the genome [8, 9]. What’s more, current technological advances in computing and analytical techniques have enabled researchers investigating evolutionary end points to routinely incorporate large numbers of markers from hundreds of individuals, to gain insight into demographic processes [8, 10, 11]. An exemplary instance of contemporary capability makes use of the butterfly, *Heliconius melpomene*, which has benefited from high-quality genome assemblies used to resolve population structure, and is underpinned by wing colour [12, 13]. While population-specific differentiation at genomic regions has enabled the identification of mechanisms controlling phenotypic variation, it has also been found that these traits have instigated speciation [14]. As such, local adaptation (as reviewed by Savolainen et al. [15]) can be used to distinguish adaptive mechanisms in populations and can effectively complement species-level comparisons to not only define evolutionary commonality, but also to provide insight into traits and processes that have driven speciation.

Defining genomic variation associated with local adaptation and speciation is particularly pertinent to the ecotoxicological model earthworm, *Lumbricus rubellus*. This species is a major terrestrial sentinel that consists of highly divergent lineages [16, 17] and shows evidence of forming discrete populations across the UK [18]. Its use as a model is, in part, due to the relative sensitivity of *L. rubellus* to contaminants, persistent contact with upper-most region of soil and important ecological role in nutrient cycling. Much of the research that uses *L. rubellus* has focussed upon the effects of heavy metal contamination, and a body of work has built up upon understanding how this species is capable of persisting across highly contaminated former mine sites [19–21]. The relatively high reproductive rate of many invertebrates enables rare or novel variants associated with adaptive properties to spread quickly throughout populations under significant selection pressure, and many invertebrates have been found to have genetic bases for adaptation to metal contaminants [22–25]. Resistance to metal contamination has been suggested in populations of *L. rubellus*, compared to those inhabiting comparatively clean soils [19–21], though few inferences have been possible concerning the potential mechanisms involved.

Understanding adaption in *L. rubellus* is complicated because this species is comprised of a number of highly diverse lineages. Across continental Europe, several mitochondrially divergent lineages have been recorded [26], with *L. rubellus* appearing to be a highly polymorphic species. Alternative analyses focussing on British earthworm lineages have found evidence for behavioural drivers of isolation, postulated to be the result of lineage-specific pheromones [27]. While very little data has been able to distinguish drivers of speciation between the two lineages found throughout the UK [16, 28], it is still unknown as to whether lineages, and therefore potential adaptive mechanisms are independent.

Here, we used RADseq with the aim of identifying the impacts of selection upon genetic variation in populations of *L. rubellus* previously identified at sites that have been historically exposed to heavily contaminated mine soils. Specifically, we defined the discrete nature of earthworm populations at the lineage and at the local level, putting emphasis upon identifying genetic variation that segregates populations relating to soil pollutants at highly contaminated mine sites. Initially, we made use of draft reference genomes from both lineages present in the UK to infer lineage specific associations, thereafter inferring population structure to determine whether any similarities exist in the relative ability of certain genotypes to inhabit specific soil types. Through comparison with proximal populations found on less contaminated soils, we have provided insights into ecological processes derived from segregating patterns of genetic differentiation, while calculation of population genetic statistics will demonstrate the long term effects of environmental contamination upon natural populations. Finally, access to genomic scaffolds has allowed us to determine proximal genes that are likely under selection and therefore surmise adaptive mechanisms and pathways that are shared between lineages.

## Methods

### Collection

**Fig. 1:**
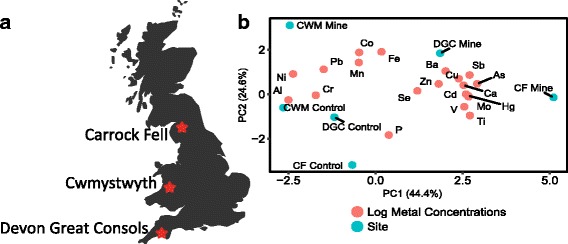
Locations across the UK where *L. rubellus* were collected (**a**) and a PCA of log metal concentrations demonstrating comparable site soil characteristics for each of the sites (**b**). Chemical symbols are used to refer to specific metals and the amount of variation explained by PC1 and PC2 is 44.4% and 24.6%, respectively. Site name abbreviations are: Carrock Fell (CF), Cwmystwyth (CWM) and Devon Great Consols (DGC)

Earthworms were sampled in 2010 from three UK sites with known histories of metal pollution as well as local control sites with low soil metal concentrations. The three sites were Devon Great Consols (DGC, *n* = 40), a former As and Cu mine in Devon, South West England; Carrock Fell (CF, *n* = 29) an As and W mine located on the edge of the Lakes District of North West England, and also from Cwmystwyth (CWM, *n* = 59), which is a former Pb mine located in Mid-Wales. Sample site coordinates are reported in Additional file 1: Table S1 and the general site location in the UK is demonstrated in Fig. 1a, which was made in R [29]. *L. rubellus* lineages were sampled from sites using a fork to dig into the epigeic (top 10 cm) of soil, before individuals were removed by hand. After collection, all earthworms were maintained upon native soils until returned to the laboratory. Individuals were rinsed with deionised water and a sample from their posterior was taken using a sterile scalpel blade. Both tail segments and remaining tissues were then frozen in liquid nitrogen and stored at -80 ^o^C for latter processing.

### Soil metal quantitation

Approximately 5 g of soil were removed from three replicates from each site where *L. rubellus* was collected. Soils were dried for 48 h at 80 °C before being passed through a 2 mm sieve. Soils were analysed for total soil metal concentrations as described in [30]. Briefly, following aqua regia digestion using a microwave system, samples were quantified using a Perkin Elmer Elan DRCII inductively coupled plasma mass spectrometer (Perkin Elmer 4300DV). Quality control within the analyses was conducted using the standard reference material ISE 192 (International Soil Exchange, The Netherlands). Values were log transformed prior to PCA using Minitab, before results were plotted in R v. 3.1.2 [29] using ggplot2 v. 1.0.1 [31].

### RADseq library preparation

DNA was extracted from samples using Qiagen blood and tissue kits with RNase A, and quantified with a Qubit 1.0. RADseq libraries were derived from these high quality DNA samples by following the protocol initially published by Etter et al. [32], with some modifications, detailed in Additional file 2. The method works to provide thousands of markers by selectively amplifying restriction sites that are ligated to adapter sequences, each containing primers for high throughput sequencing. Briefly, samples were digested with SbfI, before a P1 adapter was ligated and samples pooled. Samples were then randomly sheared using a Covaris S series (Brighton, UK) applying a regime to achieve 300–800 bp fragments, and P2 adaptors ligated. Libraries were assessed for quality using qPCR before being sequenced on a HiSeq 2000 by the GenePool Laboratory (Edinburgh, UK).

### Read processing

Raw sequence data was assessed for quality and processed using Stacks v. 1.30. Briefly, process_radtags was used to demultiplex samples, remove low-quality reads and trim to 90 bp. All samples achieved greater than 100,000 reads following this process, with the average being 2.083 M reads. Reads were aligned to genomes sourced from *L. rubellus* lineages A and B, representing the two cryptic lineages found in the UK, using BBMap v. 35.51 (http://sourceforge.net/projects/bbmap/). Only reads that aligned uniquely were used in subsequent analyses. Reads aligned to the lineage B genome (composed of 175,919 scaffolds, N50 = 9581) were forwarded to the ref_map package from Stacks, which was run using the options –n 3 and –m 3. The Populations module of Stacks was then run, limiting the output to loci existing in at least 10% of the population with at least 5× coverage, resulting in 219,545 SNPs. The Populations module outputs SNP data in Plink and Structure formats, limited to a single SNP per locus, chosen at random to account for linkage. Inbreeding coefficient and neucleotide diversity were calculated by the populations module of Stacks, while the “--missing” option in Plink v. 1.90b3.29 [33] was used to determine the amount of missing data in each individual. To specifically search for markers near pheromones not identified in the analysis, we used tBlastn [34] to define candidate scaffolds from the lineage B genome that contained genes incorporated into the analysis of pheromones by Novo et al. [35]. We then reran the populations module for all markers aligning to these scaffolds to assess genetic variation between sites.

### Population structure

The smartPCA module of EIGENSOFT v. 6.0.1 [36] was used to perform principle component analysis (PCA) in order to determine the membership of individuals to specific population clusters. No automatic outlier removal was allowed and a Tracy-Widom distribution was used to infer statistical significance. To determine lineage membership of individuals, we calculated the correlation between principal components for all individuals and the proportion of reads aligning to lineage specific genomes was calculated using Pearson’s correlation coefficient in Minitab v. 17. In addition to this, the software, Structure v. 2.3.4, was used to implement a model-based clustering method for determining population structure and assign individuals to *K* populations [37, 38]. For all runs, an initial run of 1000 burn-in followed by 1000 repetitions where data was collected, with *K* = 1, was used to estimate the allele frequency distribution (lambda). The distinction between lineage A and B (*K* = 2) was characterised with an initial run of 100,000 burn-in followed by 100,000 data collection. Individuals were split into two groups, representing each of the two lineages, and structure was run again to test for the number of discrete populations therein, testing across runs (50,000 burn-in, 50,000 data collection) implementing values of *K* from 1 to 7 with 10 replicates of each. Structure harvester was used to identify the most appropriate value of *K* via implementation of the Evanno method [39, 40]. The value of *K* where delta *K* is highest was used in a final run (100,000 burn-in, 100,000 data collection) that was plotted using Distruct2.pl (http://www.crypticlineage.net/pages/distruct.html). The pairwise relationships of all individuals found locally to each other were formally assessed using KING kinship coefficient estimator v. 1.4 [41], which was also implemented for multidimensional scaling (MDS).

### Population genetic statistics and outlier analysis

After binning individuals in populations as defined by population structure analyses, we reran the Populations module of stacks to determine lineage and site-specific population genetic end points, which was also used to derive Fisher’s exact test results. One-way ANOVA in Minitab v. 17 was used to determine significant differences for nucleotide diversity and inbreeding coefficient, between lineages and the contamination status of sites. Bootstrapped 95% confidence intervals were calculated using the scikits module (https://github.com/cgevans/scikits-bootstrap#egg=Package) in python 3.4, with a bootstrap sample pool of 20,000. To define markers, or associated regions, that are under selection, we used statistical tools for detection of outlier SNPs that deviate from those that are neutrally evolving. PGDspider v. 2.1.0.0 [42] was used to convert data to genepop format before Bayescan v2.1. [43] was run under default settings. Outlier SNPs found to be under selection were independently verified using PCAdapt, where principle components (K), ranging from 1 to 15 were initially compared using a “scree plot”. A value of K = 10 and a stringent false discovery rate (alpha = 0.001) were implemented so as to impose a strict filter for SNPs considered to be under selection. Scaffolds surrounding the mapped genomic location of SNPs were extracted from the draft *L. rubellus* lineage B genome using biopython scripts (http://biopython.org/). These regions were checked against the nr database using blastx from Blast + v. 2.2.29 [34] to Identify candidate genes likely to be under selection. Generalised annotations and GO terms were ascribed using BLAST2GO [44].

## Results

### Site soil metal characterisation

Soil metal concentrations that were used to characterise the sites from which earthworms in this analysis were collected (Additional file 1: Table S2). A PCA of the results shows that control soils are more highly correlated with each other than to any of the mine sites (Fig. 1b). Specifically, the mine site at CF is characterised as having greater levels of Cd, Hg, V, Ti and Mo than other sites, but shared high levels of As and Cu with the mine site at DGC. The mine site at CWM has particularly high levels of Pb and Mn, relative to the other sites surveyed. These results signify that earthworms inhabiting soils at former mine sites are likely under different selection pressure relative to each other.

### Sequencing output and lineage identification

Sequencing resulted in a total of 266.8 M reads, with an average of 2 M reads per sample. RADseq of 128 samples resulted in a Stacks catalogue of 4,527,370 loci containing SNPs, with an average coverage of 12.2 reads for each loci added to the catalogue. 219,545 SNPs were maintained after filtering via the populations module of stacks and subsequently used in population genomic analyses. The amount of missing data per individual, averaging 92.8%, is listed for each sample in Additional file 1: Table S3, which also details sequencing, coverage and alignment results.

**Fig. 2:**
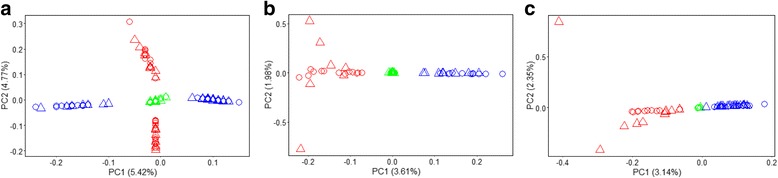
Principle component analyses of RADseq data for populations of *L. rubellus* collected across the UK, representing all individuals (**a**, *n* = 128), those from lineage A (**b**, *n* = 66) and Lineage B (**c**, *n* = 62). Triangles represent individuals originating from former mine sites, while circles represent those from nearby control sites. The amount of variance explained by each component is noted on their respective axes

**Fig. 3:**
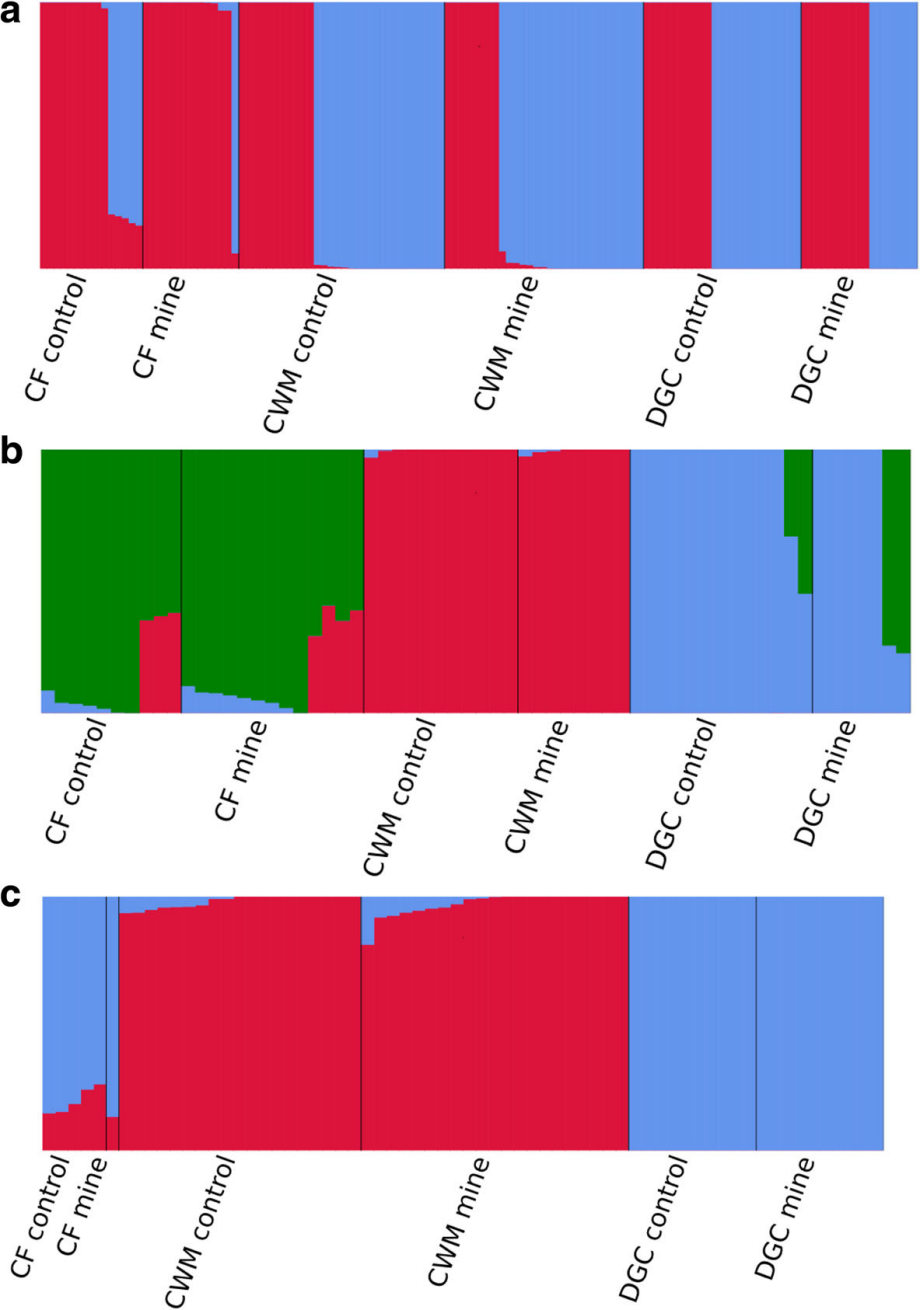
Structure results from RADseq data for *L. rubellus* collected from former mine sites and nearby control sites across the UK. Each bar represents an individual, with the proportion of colours reflecting affiliation with differentiated populations. **a** represents all individuals sampled (n = 128), where K = 2, and highlights the distinctions between lineages A and B, coloured red and blue, respectively. **b** and **c** demonstrate the best supported clustering of individuals belonging to lineage A (*n* = 66, K = 3) and B (*n* = 62, K = 2), respectively

We used PCA to provide initial insight into the structure of *L. rubellus* populations from SNPs derived from RADseq. The first 5 PCs were each significant (*P* < 1 × 10^−12^) via the Tracy-Widom statistic, when considering all individuals even though each explains only a relatively small proportion of overall variance; though consistent with similar analyses [45, 46]. Plotting PC1 and PC2 (Fig. 2a), representing 5.42% and 4.77% respectively, shows individuals forming two clearly discrete clusters for each CF, CWM and DGC. Individuals from CF tend towards the centre of the plot and separation between two clusters is less obvious. PC3 also appears to distinguish samples by collection site (Additional file 1: Figure S1), whereas PCs 4 and 5 mainly discriminate between individuals from DGC. Values of K used to determine the likely number of population clusters as defined by Structure were derived using the evanno method and are listed in Additional file 1: Table S4. A value of K = 2 was strongly supported when considering all samples (Fig. 3a), which were then binned in concordance with clusters formed in the PCA. Pearson’s correlation coefficient was used to determine whether or not the proportion of reads aligning to lineage specific reference genomes (Additional file 1: Table S3) corresponded with genomic variation associated with population structure, subsequently finding that the first 2 PCs were significantly, positively correlated (*p* < 0.05, Pearson’s correlation coefficient = 0.76 and 0.32 for PC1 and PC2, respectively). Individuals assigned to lineage B possessed a far greater proportion of reads aligning to the lineage B genome (21–43%) over those assigned to lineage A (<12%). Under these assignments, individuals from CF had the highest skew towards a single lineage, with only a single individual from contaminated soils assigned to lineage B (blue), while 27.3% of genotypes attributed to the 5 lineage B individuals from the control population clustered with lineage A (red). Net nucleotide distance between the lineages, as calculated in structure, was 0.126.

### Population structure among lineages

For both *L. rubellus* lineages, we used PCA to highlight the numbers of discrete clusters forming along significant principle components, before deriving the proportion of an individual’s genotypes that clustered together using the software, Structure. For lineage A, only the first PC was significant (Fig. 2b), with PC1, explaining 3.61% of the variance, demonstrating clusters of individuals formed through sample site. Structure analysis highlighted that 3 discrete populations best represented the data and broadly define the geographic regions from which individuals were sampled (Fig. 3b). Genotype clusters 1 (green), 2 (red) and 3 (blue) tended to define populations as CF, CWM and DGC, respectively, with individuals sampled from CWM observing the highest proportion of discrete clustering (>99% of genotypes). Net nucleotide distance ranged between 0.014 and 0.019. For earthworms in lineage B, The first 3 PCs were significant (Fig. 2c), with PC1 separating individuals based upon sample site, explaining 3.14% of the variance, whereas PC2 and PC3 (Additional file 1: Figure S2) appeared to discern variation among individuals from DGC. The structure results suggest that the data consists of 2 populations (Fig. 3c), with cluster 1 (red) representing earthworms from CWM, while CF individuals most resemble those from DGC, who are completely associated with cluster 2 (blue). Net nucleotide distance in structure was calculated as 0.19. Kinship coefficient, defined as the probability that two alleles sampled at random from two individuals are identical by descent, were plotted against the probability that the two individuals share zero alleles identical by state (Additional file 1: Figure S3). No individuals are considered to be related (i.e. closer than 3rd Degree relatives, e.g. first cousins), while 2 pairs of individuals from DGC are the most related out of any individuals assessed. In CF, Lineage B individuals are generally more related than lineage A individuals. An MDS plot that makes use of identity by state and is able to distinctively separate the lineages on dimension 1 and relative to site on dimension 2, while individuals from DGCC and DGCM cluster relative to dimension 5, regardless of lineage (Additional file 1: Figure S4).

### Population genetic results

**Fig. 4:**
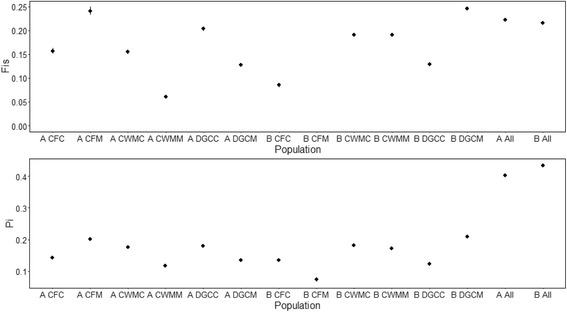
Lineage-specific summary genetic statistics in populations of *L. rubellus* from control and mine sites, showing inbreeding coefficient (F_*IS*_, Top) and nucleotide diversity (Pi, Bottom). Error bars represent standard error. No marker for F_*IS*_ was calculated at “B CFM”, as this represents a single individual

Broadly, there was no significant difference (One-way ANOVA, *P* > 0.05) in the statistics defining inbreeding or nucleotide diversity that was specific to lineage or the contamination status of sites, at least with regard to the environmental factors considered (Fig. 4). Unexpectedly large differences in allele frequency are likely to be symptomatic of SNPs associated with regions of the genome that are under selection and can be used to define key differences between populations and lineages. Using Bayescan, we observed 982 SNPs significantly associated with differences between the two lineages, found to have a false discovery rate (q-value) under 0.05, and are therefore considered to be under selection Additional file 1: Figure S5a). Evidence for selection is demonstrated via log posterior odds scores (PO), with higher scores demonstrating a higher likelihood of selection. 50 SNPs underwent further analysis having achieved a log_10_PO > 1.96. These SNPs occur over 49 separate scaffolds of the lineage B assembly and have been explicitly annotated in Additional file 3. As the power of Bayescan to provide accurate results in the presence of hierarchical population structure or admixture can result in false-positives, we used PCAdapt to independently support the analysis of SNPs under selection. Based upon a “scree plot” that defined the amount of variation explained by principle components (K) up to 15 (Additional file 1: Figure S6), we used a K of 10 and a false discovery rate of 0.1% to identify 8115 outlier SNPs. Of these, four SNPs of those with the top 50 log_10_PO scores identified by Bayescan weren’t included and are specified in Additional file 4.

The most significantly differentiated SNP defining lineages occurs at a site proximal to a gene affiliated with histone lysine demethylation and is therefore a major regulator of chromatin structure (Table 1) [47]**.** Annotation of scaffolds where the other top candidate SNPs are located shows that four contain genes affiliated with collagen, a protein that is highly abundant in animal tissues as the main component of connective tissue [48].

**Table 1:** Annotation results output by blastx for SNPs considered most significantly under selection between lineages A and B

Marker	Scaffold name	Sequence length (bp)	Sequence description	Minimum e-value	Bayescan log10(PO)
1	scaffold694	35,309	lysine-specific demethylase 4A isoform X3	2.72E-55	3.2215
2	scaffold626	36,160	collagen alpha-1(I) chain-like isoform ×1	5.99E-10	2.8532
3	scaffold84914	3222	---NA---		2.8532
4	scaffold1008	32,106	syntaxin-binding 1-like isoform ×1	4.55E-16	2.7951
5	scaffold486	38,320	PREDICTED: uncharacterized protein LOC109476627	5.06E-46	2.7951
6	scaffold4171	29,021	keratin-associated 12–2-like	6.00E-08	2.7439
7	scaffold55819	5250	potassium voltage-gated channel Shab-like	3.73E-60	2.6187
8	scaffold14485	12,918	ubiquitin thioesterase	4.87E-11	2.5838
9	scaffold14102	13,094	hypothetical protein HELRODRAFT_80699	2.16E-05	2.5215
10	scaffold20298	10,806	probable G- coupled receptor No9	2.07E-67	2.4185

The e-value refers to the expected number of random hits for an alignment by blastx, with lower values being more significant matches. Evidence for selection is demonstrated by the log posterior odds scores (PO), with higher scores demonstrating a better likelihood of selection than no selection. All SNPs are considered as candidates under selection by Bayescan and PCAdapt. A complete table can be found in Additional file 3

Several scaffolds were found to be likely bearers of pheromones, therefore, those where the top hit achieved an E-value greater than 1 × 10^−19^ were included, reflecting a highly significant match to the results available to the database used by blastx. A SNP for proximal to Temptin5 was found to segregate almost entirely by lineage (F_*ST*_ = 0.992), with the marker found to be missing in individuals from lineage A, except for one that was heterozygous and another that was homozygous for the alternative allele.

In our analysis of lineage-specific genetic variation associated with populations inhabiting highly contaminated former mine sites, no single SNP was found to segregate any of the mine sites from their respective control sites and no outliers were considered to be under selection across any of the sites assessed (Additional file 1: Figure S3 b-f). SNPs with a nominal Fisher’s exact test *P*-value <1 × 10^−5^ were analysed to determine the functionality of proximal genes. A number of SNPs are associated with scaffolds containing genes for metal binding or transport, as well as stress responses, and are candidates for further analysis regarding biochemical mechanisms associated with detoxification (Table 2, Additional file 3).

**Table 2:** Lineage-specific annotation results for scaffolds containing SNPs most significantly differentiating populations of *L. rubellus* inhabiting contaminated former mine sites and nearby control sites

Marker	Scaffold name	Sequence length (bp)	Sequence description	Minimum e-value	P-value	Site	Lineage
1	scaffold596	38,917	PREDICTED: uncharacterized protein LOC105439557	1.00E-114	2.44E-07	CWM	A
2	scaffold90812	2919	---NA---	–	1.05E-06	CWM	B
3	scaffold51697	8321	glutamate receptor 1- partial	4.90E-01	1.55E-06	CWM	A
4	scaffold114081	3538	ubiquitin thioesterase partial	1.50E-09	1.74E-06	CWM	A
5	scaffold34161	7815	techylectin- partial	2.00E-26	2.30E-06	CWM	A
6	scaffold7091	27,562	organic cation transporter protein isoform x1	1.60E-06	2.98E-06	CWM	A
7	scaffold65438	4451	potassium voltage-gated channel subfamily a member 3-like	0.00E + 00	6.66E-06	DGC	B
8	scaffold83338	3307	polypeptide n-acetylgalactosaminyltransferase-like partial	8.40E-09	7.42E-06	DGC	B
9	scaffold53218	5489	membrane metallo-endopeptidase-like partial	4.60E-11	7.94E-06	DGC	B
10	scaffold111638	2040	PREDICTED: uncharacterized protein LOC106143484	2.00E-39	7.94E-06	DGC	B

SNPs were included when P < 1 × 10^−5^, as calculated using Fisher’s exact test by the Populations module of Stacks. The e-value refers to the expected number of random hits for an alignment by blastx, with lower values being more significant matches. A complete table can be found in Additional file 3

The lowest *P*-value (*P* = 2.44 × 10^−07^) was observed in lineage A individuals from CWM on a scaffold that sees two SNPs nearby to a PARP gene, involved in DNA repair and programmed cell death. Also on this scaffold is a predicted palmitoyltransferase gene (*ZDHHC3*), known to mediate Ca2+ transport across cell membranes [49]. Far fewer SNPs in earthworms from DGC and CF were found to segregate populations at mine and control sites. For lineage B individuals from DGC, a phosphate membrane transporter is significantly implicated (*P* = 6.66 × 10^−6^) in defining individuals between contaminated and control sites, pertinent to the transport of the phosphate analogue, arsenate [50, 51]. Another segregating SNP that has significant implications (*P* = 2.58 × 10^−5^) lies near to a metallothionein that has previously been identified in *L. rubellus* [52] and is synonymously identified among lineage A individuals.

## Discussion

Here, we have principally demonstrated that we can observe population structure in *L. rubellus* using a high-density set of SNPs, and are able to clearly distinguish between lineages, which tend to form discrete populations across the UK in most instances. Subsequently, we have demonstrated that populations found on highly contaminated former mine sites are almost indistinguishable from those from nearby, relatively unpolluted soils, except for at a small number of sites across the genome. Many of the most significantly different genetic signatures that segregate between populations and species allow us to gain further insight into mechanisms playing a role in local adaptation and species divergence.

Firstly, we were able to distinguish between lineages through alignment to two voucher draft genomes and make clear genomic distinctions between the two lineages. Giska et al. [17] demonstrated that individuals sampled throughout continental Europe clustered relative to sampling location when assessing nuclear markers derived from RADseq, rather than by mitochondrial lineage. However, these authors also demonstrated that the two lineages extant in the UK were the most divergent of all lineages compared using mitochondrial sequences. We find that populations cluster primarily by lineage, even across broad geographic distances, and are supported by Donnelly et al. [53], who used microsatellites. In this instance, genotypes relating to lineage across the 3 sites appear to be characterised, almost entirely, as a single lineage, with the exception of lineage B individuals from CF who display a small proportion of genotypes characterised under lineage A. The proportion of the genotypes clustering with lineage A (red) in the structure results is, however, small enough to not be considered recent or ongoing, while we see little evidence of reciprocal gene flow. What’s more, the PCA and MDS differentiate lineage A individuals from CF as a single, discrete population. Recent work by Dupont et al. [54] has suggested that hybridisation, particularly with respect to unidirectional gene flow, might be responsible for a similar disparity recorded among populations of *Aporrectodea caliginosa* that were surveyed using nuclear and mitochondrial markers. Individuals at CF demonstrate relatively low *FIS*, while subsequent kinship analysis shows that no individuals from CF were as, or more related, than first cousins. We provide further evidence against hybridisation through use of MDS, which incorporates identity by state to clearly differentiate between lineages across the first dimension. While there is mixed evidence both for and against population structure and gene flow across smaller spatial scales [55, 56], such as that between mine sites and their respective control sites assessed here, our inferences are well-supported and provide a basis for future work.

In our analysis of SNPS most significantly segregating between the two lineages, a number occur close to genes implicated in processes that have recently been suggested as drivers for lineage differentiation. The most well supported SNP was proximal to a gene controlling histone lysine demethylation, which is one of the most prominent epigenetic mechanisms controlling chromatin structure. DNA methylation is another epigenetic modification that has previously been differentiated among *L. rubellus* lineages at DGC in an analysis by Kille et al. [20] . In this work, the authors incorporated AFLPs and methylation sensitive AFLPs to demonstrate aspects of population structure relative to the arsenic burden of soils, with individuals belonging to lineage A appearing to diverge based upon genomic data, and those considered to be from lineage B appearing to be structured based upon variation at methylated sites. Our findings here further suggest genomic variation among the epigenetic machinery and any further work would benefit from marrying novel accessibility to what is essentially a single cell type in the chloragosome, with new techniques linking transcriptomic variation with that in the epigenome to explore the adaptive plasticity in each lineage.

Work by Jones et al. [57] suggests that there is behavioural variation driving reproductive isolation between the British lineages, which builds from the discovery that two distinct types of pheromone were previously found in the *L. rubellus* genome [58]. Indeed, when analysed in this instance, we found substantial evidence for segregation between the lineages at a site proximal to a temptin gene, which encodes a water-borne sex pheromone previously found in earthworms [35]. Overall the evidence provided here suggests that the two lineages are essentially discrete within the UK, but can’t entirely discount non-recent admixture. It is, of course, possible to observe sites associated with incomplete lineage sorting using techniques such as the *D* statistic [59], though this will require better phylogenetic resolution across the lineages and possibly in closely related species.

Soil metal concentrations, both here and in the literature, have demonstrated concentrations of metals at these sites known to be well above those known to cause effects on naïve populations [20, 21, 30, 60–62]. It is, therefore, reasonable to suspect that the populations at these sites would be negatively affected or show signals of selection when compared to earthworms from control sites, though we observe little variation symptomatic of bottlenecks among populations inhabiting mine sites. There is no obvious definition in population structure between mine or control sites, reflecting that populations are most likely the result of genomic variation present within the background of control populations. Though there appears to be little differentiation between sites other than this, we are able to use contemporary analytical tools to identify segregating SNPs and signals of selection.

CWM is the site of a former Pb/Zn mine that discontinued production before 1920 [63], and though we see similar levels of Pb at each of the sites surveyed, previous work at the site (known as CWM Stream) found it to have far higher free ion concentrations than any other sites sampled [63, 64]. In our analyses, a number of markers proximal to genes associated with calcium ion management and DNA damage repair mechanisms were found to segregate between earthworms found at the two sites, befitting with previous research [65, 66]. A study by Andre et al. (2010), implicated the role of genes in the Ca-signalling pathway to management of Pb in earthworms from CWM, before describing variation near a gene encoding an intracellular Ca-transporter gene, *SERCA*, that varies between individuals identified as lineage A and a control population. A number of the genes identified during this investigation purport to this hypothesis and are therefore of likely importance to earthworm continuation at this site in both lineages.

Evidence supporting adaptation of *L. rubellus* from DGC and CF to As has been previously demonstrated [19, 20] and is supported with biochemical data focussing upon As speciation and implicates specific detoxification pathways. Following exposure, the proportion of arsenate and arsenobetaine has been found to decline in body concentration, while the proportion of arsenite increases [67, 68], likely reflecting the reduction of arsenate by arsenate reductase [69]. Genes, such as this, which associated with handling and detoxification of the most common arsenic species, arsenate and arsenite, are well described [70–72] and relate to analogous phosphate mechanisms and chelation to prevent protein degradation, respectively [73]. In earthworms from DGC, genetic variation near a phosphate channel can implicate an effort to affect intracellular concentrations of arsenate. We also see variation associated with a metallothionein, previously identified in *L. rubellus* [52], which would likely be in response to elevated arsenite, binding via sulphydryl groups [74]. Synthesis of metallothioneins is closely associated with that of phytochelatins, which have been found to increase relative to arsenate exposure in the laboratory and likely bind to arsenite [68].

Our analysis is likely to be underpowered in our ability to distinguish specific SNPs, particularly for lineage B individuals from CF where we see no major variants. However, selection pressure across sites can vary immensely, given the highly heterogenous nature of soils; therefore, attempting to define the effects of large-scale contamination may not be as simple as detecting resistance for a single toxicant. Work by Spurgeon et al. [16] and Liebeke et al. [28] has investigated environmental and metabolomic variation in *L. rubellus* across the UK, in attempts to observe drivers for lineage divergence. Specifically, Spurgeon et al. [16] found that soil pH and the percentage of organic matter correlated with the proportion of earthworms from lineage A at sites, while tissue As accumulation was also variable between lineages. Here, we find that genes associated with mechanistically similar processes, in particular calcium movement in earthworms from CWM and metallothionein in those from DGC, are shared between lineages. Though we’ve demonstrated population-specific variation around genes relating to managing toxicants, we observe convoluted signals that populations of *L. rubellus* originating from mine sites are perturbed in the long term. The fact that we fail to resolve population structure between mine and control sites, or to even see complete segregation at any SNPs, demonstrates that earthworms inhabiting highly contaminated former mine sites are likely derived from standing genetic variation extant among populations in less contaminated soils and may even continue to experience gene flow.

## Conclusion

We’ve used genomic analysis of earthworms in the UK with the view of identifying genomic variation in response to environmental variables. While we can observe population structure and define lineages, it is difficult to resolve markers that segregate entirely between populations in response to soil metal concentrations. This may represent a highly variable series of traits in response to the heterogenous nature of the soil environment, but ultimately demonstrates that natural populations of a cosmopolitan species are generally robust to long term metal contamination. This system demonstrates the importance of determining ecological end points of ecotoxicological models, as their relevance can shift relative to population and evolutionary history. Beyond this, *L. rubellus* appears to provide an exemplary system for exploring drivers for speciation, with a continuum of lineages coexisting across continental Europe, while distinct lineages exist in isolation throughout the UK. Further work with this species will provide evidence as to the effect of genetic diversity upon adaptive capability.

## Additional files

Additional file 1: Figure S1. Significant principle components (PC3–6) defined by the Tracy-Widom statistic, calculated from RADseq data of all *L. rubellus* (*n* = 128) sampled across from the UK. Triangles represent individuals originating from former mine sites, while circles represent those from nearby control sites. The amount of variance explained by each component is noted on their respective axes. **Figure S2.** Principle component analysis remaining significant PCs, calculated from RADseq data of populations of *L. rubellus* belonging to lineage B (*n* = 66). The amount of variance explained by each component is noted on their respective axes. Circles represent control sites, triangles represent former mine sites. **Figure S3.** Intra-population estimation of kinship coefficient among *L. rubellus*, relative to lineage and sample site. A negative kinship coefficient estimation indicates an unrelated relationship. **Figure S4.** Plot of multidimensional scaling analysis incorporating IBS for populations of *L. rubellus*, relative to lineage and sample site. The first 6 dimensions are reported and the amount of variance explained by a particular dimension is detailed on their respective axis. **Figure S5.** Outlier results as calculated by Bayescan when a q-value (false discovery threshold) of 0.05 is imposed. Each SNP is plotted to infer signals of selection when populations are compared, including lineages A and B (a), as well as lineage-specific populations inhabiting former mine sites and proximal control sites (CF-A, b; CWM-A, c; DGC-A, d; CWM-B, e; DGC-B, f). **Figure S6.** Scree plot for the proportion of variance explained by principle components 1–15 as determined by PCAdapt. (DOCX 447 kb)

Additional file 2: RADseq Protocol. An explicit description of the RADseq protocol, listing the methodology, reagents and equipment for amplifying and sequencing genomic libraries with an Illumina HiSeq. (XML 63 kb)

Additional file 3: Gene annotation data output by blastx as associated with SNPs considered to be most strongly under selection. Tab 1 lists annotations for 49 scaffolds containing SNPs differentiated between lineages A and B. Tab 2 lists annotations for 39 scaffolds containing SNPs differentiated between earthworms found on former mine sites and proximal control sites. (XML 80 kb)

Additional file 4: Lineage B genome locations of all SNPs, including respective values output by Bayescan, PCAdapt and Fisher’s exact test when comparing populations for signals of selection. (ZIP 479 kb)

## Abbreviations

AFLPs: Amplified fragment length polymorphisms
bp: base pairs
CF: Carrock Fell
COII: Cytochrome oxidase II
CWM: Cwmystwyth
DGC: Devon Great Consols
MDS: Multidimensional scaling
mtDNA: Mitochondrial DNA
PC: Principle component
PCA: Principle component analysis
PCR: Polymerase chain reaction
qPCR: Quantitative PCR
RADseq: Restriction-associated DNA sequencing
SNP: Single nucleotide polymorphism
UK: United Kingdom
