# Supplementary material for: Genetic variation in populations of the earthworm, Lumbricus rubellus, across contaminated mine sites
Source: BMC Genet. 2017 Nov 17;18:97. doi: 10.1186/s12863-017-0557-8 (PMC5693503; doi:10.1186/s12863-017-0557-8)
Supplement: Supplementary file 1 — Significant principle components (PC3–6) defined by the Tracy-Widom statistic, calculated from RADseq data of all L. rubellus (n = 128) sampled across from the UK. Triangles represent individuals originating from former mine sites, while circles represent those from nearby control sites. The amount of variance explained by each component is noted on their respective axes. Figure S2. Principle component analysis remaining significant PCs, calculated from RADseq data of populations of L. rubellus belonging to lineage B (n = 66). The amount of variance explained by each component is noted on their respective axes. Circles represent control sites, triangles represent former mine sites. Figure S3. Intra-population estimation of kinship coefficient among L. rubellus, relative to lineage and sample site. A negative kinship coefficient estimation indicates an unrelated relationship. Figure S4. Plot of multidimensional scaling analysis incorporating IBS for populations of L. rubellus, relative to lineage and sample site. The first 6 dimensions are reported and the amount of variance explained by a particular dimension is detailed on their respective axis. Figure S5. Outlier results as calculated by Bayescan when a q-value (false discovery threshold) of 0.05 is imposed. Each SNP is plotted to infer signals of selection when populations are compared, including lineages A and B (a), as well as lineage-specific populations inhabiting former mine sites and proximal control sites (CF-A, b; CWM-A, c; DGC-A, d; CWM-B, e; DGC-B, f). Figure S6. Scree plot for the proportion of variance explained by principle components 1–15 as determined by PCAdapt. (DOCX 447 kb) [file 12863_2017_557_MOESM1_ESM.docx]

Supplementary figures

Fig S1 Significant principle components (PC3-6) defined by the Tracy-Widom statistic, calculated from RADseq data of all *L. rubellus* (*n*=128) sampled across from the UK. Triangles represent individuals originating from former mine sites, while circles represent those from nearby control sites. The amount of variance explained by each component is noted on their respective axes.
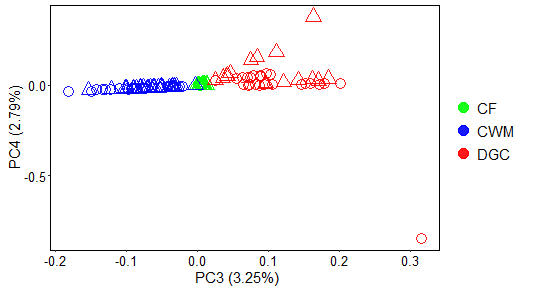

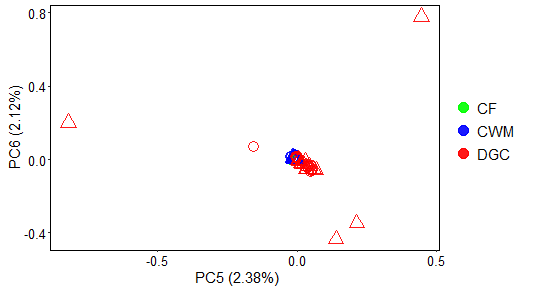


Fig S2 Principle component analysis remaining significant PCs, calculated from RADseq data of populations of *L. rubellus* belonging to lineage B (*n*=66). The amount of variance explained by each component is noted on their respective axes. Circles represent control sites, triangles represent former mine sites.


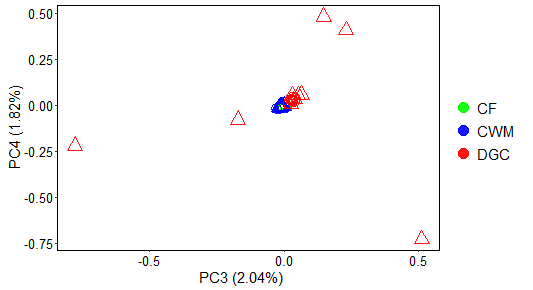


Fig. S3. Intra-population estimation of kinship coefficient among *L. rubellus*, relative to lineage and sample site. A negative kinship coefficient estimation indicates an unrelated relationship.


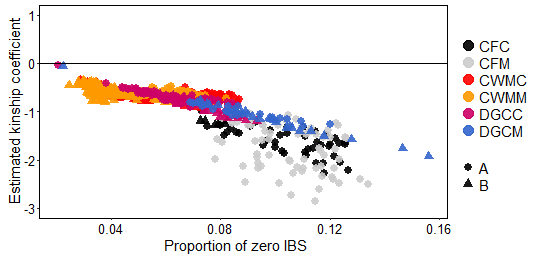


Fig. S4. Plot of multidimensional scaling analysis incorporating IBS for populations of *L. rubellus*, relative to lineage and sample site. The first 6 dimensions are reported and the amount of variance explained by a particular dimension is detailed on their respective axis.
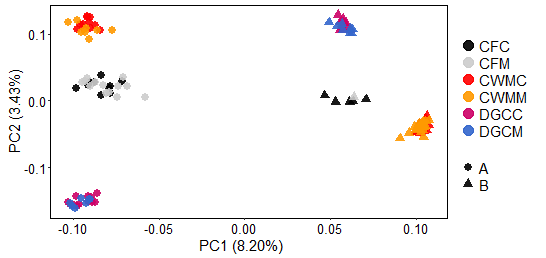

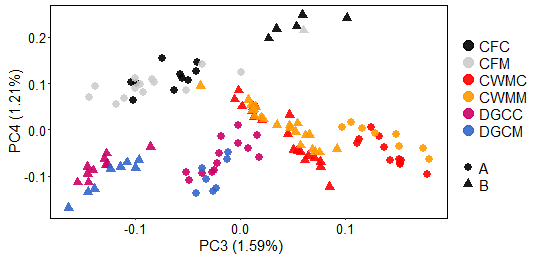

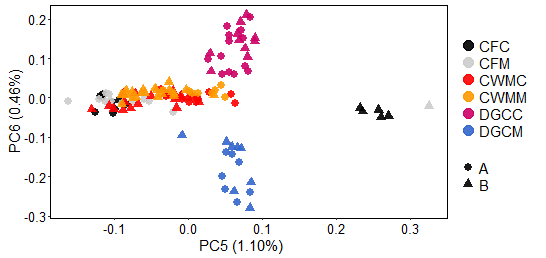


Fig. S5. Outlier results as calculated by Bayescan when a q-value (false discovery threshold) of 0.05 is imposed. Each SNP is plotted to infer signals of selection when populations are compared, including lineages A and B (a), as well as lineage-specific populations inhabiting former mine sites and proximal control sites (CF-A, b; CWM-A, c; DGC-A, d; CWM-B, e; DGC-B, f).


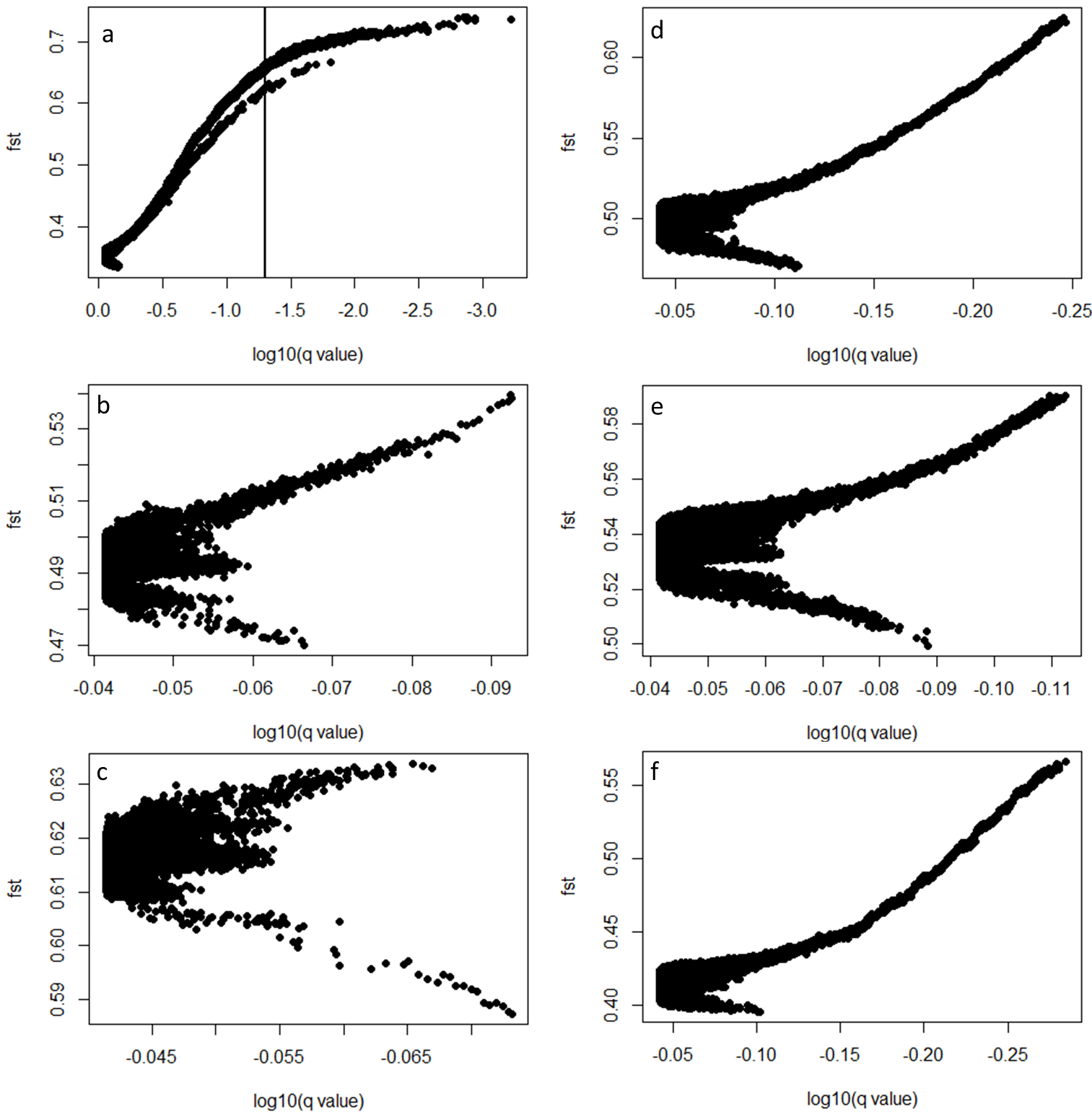


Fig S6. Scree plot for the proportion of variance explained by principle components 1-15 as determined by PCAdapt.


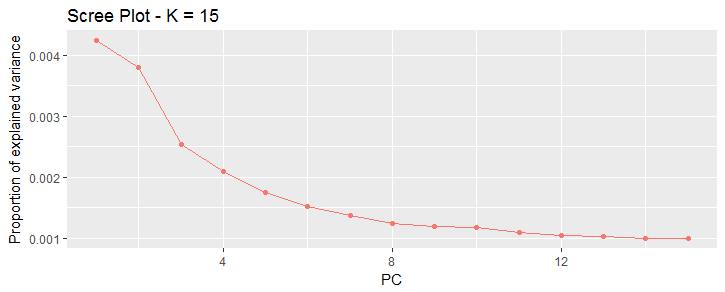


Supplementary tables

Table S1. Site details and location from where populations of the earthworm, *L. rubellus*, were collected. The number of individuals belonging to lineages A and B is also listed.

| Site Name | Soil Type | Latitude | Longitude | Lineage A individuals | Lineage B individuals |
| --- | --- | --- | --- | --- | --- |
| Carrock Fell (CF) | Mine | 54.687223 | -3.048811 | 1 | 13 |
|  | Control | 54.68016 | -3.004252 | 5 | 10 |
| Cwmystwyth (CWM) | Mine | 52.357294 | -3.761541 | 21 | 8 |
|  | Control | 52.351924 | -3.782934 | 19 | 11 |
| Devon Great Consols (DGC) | Mine | 50.53814 | -4.22322 | 10 | 7 |
|  | Control | 50.54429 | -4.222544 | 10 | 13 |

Table S2. Average soil metal concentrations (mg/kg, ± standard deviation) of sample sites from where *L. rubellus* were sampled (n=3).

|  | Site | | | | | |
| --- | --- | --- | --- | --- | --- | --- |
| Metal | CF Control | CF Mine | CWM Control | CWM Mine | DGC Control | DGC Mine |
| Al | 15200 ± 2128.4 | 11963.3 ± 2276.8 | 18166.7 ± 1415.4 | 19375 ± 434.9 | 21466.7 ± 2916 | 10126.7 ± 1726.3 |
| As | 65.5 ± 14.6 | 7290 ± 2263.8 | 20.1 ± 1 | 75.9 ± 24.6 | 315.7 ± 8.5 | 3580 ± 1361.1 |
| Ba | 49.4 ± 3.8 | 64.1 ± 23.7 | 38.8 ± 4.4 | 61.2 ± 21.8 | 47.8 ± 11.9 | 58.4 ± 16.2 |
| Ca | 735.3 ± 207.1 | 4913.3 ± 600.4 | 1237 ± 503.2 | 701 ± 356.1 | 2086.7 ± 220.3 | 6200 ± 1973.2 |
| Cd | 0.3 ± 0 | 4.8 ± 1.3 | 0.8 ± 0.2 | 0.3 ± 0.1 | 0.5 ± 0.3 | 0.7 ± 0.1 |
| Co | 5.9 ± 2.1 | 16.5 ± 3.5 | 15.5 ± 0.3 | 64.5 ± 24 | 12.8 ± 0.4 | 21.3 ± 5.3 |
| Cr | 17.1 ± 3.1 | 18.8 ± 4.3 | 23.6 ± 2.1 | 22.3 ± 1.3 | 28.2 ± 2.3 | 15.7 ± 3.3 |
| Cu | 20.2 ± 2.2 | 355.3 ± 117.6 | 47.8 ± 3.5 | 31.6 ± 4.1 | 92.9 ± 4.3 | 869 ± 209.9 |
| Fe | 33633.3 ± 5672.2 | 44600 ± 10911.9 | 38333.3 ± 2437.9 | 63200 ± 10088.9 | 43066.7 ± 1040.8 | 55733.3 ± 14785.6 |
| Hg | 0 ± 0 | 6.8 ± 0.3 | 0.1 ± 0 | 0 ± 0 | 0.1 ± 0 | 0 ± 0 |
| Mn | 728.7 ± 279.4 | 1520 ± 235.8 | 1057 ± 159.9 | 5687.5 ± 2613.6 | 642 ± 48.1 | 861.7 ± 328.4 |
| Mo | 2.9 ± 0.4 | 13.4 ± 2.5 | 0.9 ± 0.1 | 2 ± 0.4 | 1.2 ± 0.1 | 1.8 ± 0.3 |
| Ni | 9.6 ± 0.2 | 7.6 ± 2.5 | 25.8 ± 2.2 | 23.6 ± 1.5 | 21.8 ± 0.2 | 19.1 ± 3.2 |
| P | 2153.3 ± 228.1 | 1393.3 ± 259.3 | 1290 ± 252.4 | 785 ± 51.5 | 1113.3 ± 102.1 | 843.7 ± 147 |
| Pb | 136 ± 19.3 | 178 ± 56.3 | 1110 ± 36.1 | 1390 ± 301.4 | 75.8 ± 12.2 | 226 ± 29.5 |
| Sb | 1.5 ± 0.1 | 16.3 ± 5.9 | 1.5 ± 0.1 | 2.5 ± 0.4 | 1.4 ± 0.1 | 19.7 ± 5.6 |
| Se | 1.7 ± 0.2 | 1.2 ± 0.3 | 0.3 ± 0 | 1.3 ± 0.5 | 1.5 ± 0.1 | 1.9 ± 1.3 |
| Ti | 67.7 ± 10.8 | 212.3 ± 45.5 | 33.9 ± 6.9 | 14.6 ± 0.8 | 59.1 ± 16.6 | 54.8 ± 3.4 |
| V | 43.1 ± 3.6 | 104 ± 17.7 | 25.7 ± 1.6 | 29.8 ± 1.6 | 40.4 ± 3.7 | 29 ± 3.1 |
| Zn | 103.8 ± 9.2 | 431.7 ± 167.5 | 287.3 ± 28 | 158.3 ± 14 | 152 ± 36.1 | 201.7 ± 31.6 |

Table S3. Sequencing and processing statistics for individual *L. rubellus* earthworms included in this analysis. Listed are the number of raw reads and the percentage of reads aligning to both lineage A and B genomes as was reported by BBmap. Mean depth of markers for each individual, as reported by Stacks, is also included, as is the percentage of missing genotypes out of the 219,545 SNPs, as reported by Plink.

| Sample name | Number of raw reads | Mean depth of coverage for reads used by Stacks | Reads aligned to the lineage B genome | Reads aligned to the lineage A genome | Site | Contamination status | Difference in the number of reads aligning | Missing data |
| --- | --- | --- | --- | --- | --- | --- | --- | --- |
| CFC_1-8 | 227377 | 13.5 ± 16.6 | 69.4% | 58.7% | CF | Control | 10.7% | 98.4% |
| CFC_1-9 | 292315 | 15.9 ± 17.6 | 81.3% | 48.9% | CF | Control | 32.3% | 97.3% |
| CFC_2-3 | 265023 | 15.5 ± 16.3 | 85.4% | 48.8% | CF | Control | 36.6% | 97.4% |
| CFC_2-6 | 123459 | 10.2 ± 11.5 | 65.4% | 59.3% | CF | Control | 6.2% | 99.0% |
| CFC_2-7 | 373228 | 16.2 ± 25.9 | 58.6% | 51.8% | CF | Control | 6.8% | 98.2% |
| CFC_2-8 | 217884 | 13.5 ± 18.7 | 68.9% | 58.8% | CF | Control | 10.0% | 98.6% |
| CFC_3-1 | 712166 | 28.4 ± 49.3 | 69.5% | 59.6% | CF | Control | 9.9% | 97.7% |
| CFC_3-10 | 1133280 | 27.7 ± 49.5 | 45.5% | 39.2% | CF | Control | 6.3% | 97.6% |
| CFC_3-12 | 651811 | 26.2 ± 38.8 | 67.3% | 60.6% | CF | Control | 6.7% | 97.6% |
| CFC_3-13 | 126232 | 10.6 ± 10.7 | 85.5% | 51.0% | CF | Control | 34.5% | 98.3% |
| CFC_3-2 | 114060 | 10.3 ± 13.0 | 68.6% | 59.4% | CF | Control | 9.2% | 99.1% |
| CFC_3-3 | 540417 | 23.4 ± 36.6 | 69.1% | 58.9% | CF | Control | 10.2% | 97.7% |
| CFC_3-6 | 132535 | 10.4 ± 9.9 | 77.1% | 45.3% | CF | Control | 31.8% | 98.4% |
| CFC_3-7 | 483185 | 20.7 ± 36.7 | 67.2% | 59.6% | CF | Control | 7.6% | 97.9% |
| CFC_3-9 | 737422 | 30.7 ± 36.5 | 86.2% | 48.4% | CF | Control | 37.8% | 96.5% |
| CFM_1-1 | 660056 | 27.1 ± 43.6 | 70.7% | 59.8% | CF | Mine | 10.9% | 97.5% |
| CFM_1-13 | 713271 | 27.9 ± 48.6 | 68.4% | 58.7% | CF | Mine | 9.8% | 97.5% |
| CFM_1-4 | 533856 | 23.2 ± 41.1 | 68.7% | 60.5% | CF | Mine | 8.2% | 97.8% |
| CFM_1-6 | 333025 | 16.6 ± 24.6 | 68.9% | 59.0% | CF | Mine | 9.9% | 98.1% |
| CFM_1-7 | 1746906 | 54.9 ± 100.1 | 69.0% | 58.6% | CF | Mine | 10.5% | 97.0% |
| CFM_1-9 | 249791 | 12.7 ± 15.7 | 68.9% | 56.9% | CF | Mine | 12.1% | 98.3% |
| CFM_2-2 | 103687 | 9.5 ± 10.6 | 70.0% | 60.4% | CF | Mine | 9.6% | 99.1% |
| CFM_2-3 | 1636326 | 52.4 ± 87.8 | 67.5% | 57.9% | CF | Mine | 9.6% | 97.2% |
| CFM_2-4 | 475097 | 22.0 ± 31.5 | 71.0% | 59.9% | CF | Mine | 11.1% | 97.8% |
| CFM_2-6 | 975467 | 31.4 ± 53.4 | 58.5% | 50.3% | CF | Mine | 8.2% | 97.4% |
| CFM_3-10 | 146019 | 11.3 ± 14.5 | 69.1% | 59.4% | CF | Mine | 9.7% | 98.9% |
| CFM_3-4 | 292958 | 15.9 ± 17.3 | 81.9% | 46.4% | CF | Mine | 35.4% | 98.7% |
| CFM_3-7 | 591653 | 23.7 ± 38.9 | 66.9% | 59.8% | CF | Mine | 7.1% | 97.6% |
| CFM_3-8 | 109541 | 9.7 ± 11.0 | 63.5% | 54.0% | CF | Mine | 9.5% | 99.1% |
| CWMC_1-1 | 6596198 | 11.0 ± 40.2 | 87.8% | 46.0% | CWM | Control | 41.8% | 82.7% |
| CWMC_1-10 | 1295782 | 8.2 ± 15.8 | 61.3% | 52.2% | CWM | Control | 9.1% | 95.6% |
| CWMC_1-2 | 2575724 | 9.8 ± 25.7 | 64.0% | 55.2% | CWM | Control | 8.8% | 93.9% |
| CWMC_1-3 | 4813702 | 10.4 ± 33.7 | 85.4% | 44.3% | CWM | Control | 41.1% | 86.1% |
| CWMC_1-4 | 2950680 | 10.2 ± 29.1 | 65.2% | 56.1% | CWM | Control | 9.1% | 93.3% |
| CWMC_1-5 | 3291582 | 10.4 ± 29.0 | 87.5% | 45.6% | CWM | Control | 41.9% | 88.7% |
| CWMC_1-6 | 1907582 | 9.5 ± 19.8 | 87.5% | 45.7% | CWM | Control | 41.8% | 91.1% |
| CWMC_1-7 | 1580332 | 9.2 ± 20.1 | 84.9% | 45.0% | CWM | Control | 39.9% | 91.8% |
| CWMC_1-8 | 3617970 | 10.4 ± 30.7 | 88.7% | 46.8% | CWM | Control | 41.9% | 88.1% |
| CWMC_1-9 | 5013198 | 10.7 ± 32.8 | 86.7% | 45.4% | CWM | Control | 41.3% | 85.6% |
| CWMC_2-1 | 2066340 | 9.3 ± 20.9 | 84.6% | 45.3% | CWM | Control | 39.3% | 90.9% |
| CWMC_2-10 | 2134126 | 9.5 ± 23.8 | 64.6% | 55.6% | CWM | Control | 9.0% | 94.3% |
| CWMC_2-2 | 5299512 | 10.8 ± 34.5 | 87.2% | 45.9% | CWM | Control | 41.2% | 84.9% |
| CWMC_2-3 | 1688156 | 9.1 ± 20.3 | 64.4% | 55.7% | CWM | Control | 8.6% | 94.9% |
| CWMC_2-4 | 4658470 | 10.5 ± 34.1 | 58.5% | 50.9% | CWM | Control | 7.6% | 91.9% |
| CWMC_2-5 | 2793042 | 10.2 ± 33.2 | 62.4% | 54.0% | CWM | Control | 8.4% | 93.6% |
| CWMC_2-6 | 1552234 | 8.8 ± 20.1 | 63.9% | 55.1% | CWM | Control | 8.8% | 95.1% |
| CWMC_2-7 | 1132664 | 8.8 ± 14.5 | 88.8% | 46.8% | CWM | Control | 42.0% | 92.8% |
| CWMC_2-8 | 2405836 | 10.0 ± 24.4 | 87.3% | 46.6% | CWM | Control | 40.7% | 90.3% |
| CWMC_2-9 | 1666240 | 9.3 ± 19.0 | 88.6% | 46.7% | CWM | Control | 41.9% | 91.6% |
| CWMC_3-1 | 4219480 | 10.6 ± 32.8 | 84.9% | 44.9% | CWM | Control | 40.1% | 87.2% |
| CWMC_3-10 | 3501514 | 10.3 ± 28.2 | 88.9% | 47.0% | CWM | Control | 41.9% | 88.5% |
| CWMC_3-2 | 1369118 | 9.2 ± 18.1 | 86.8% | 46.1% | CWM | Control | 40.8% | 92.4% |
| CWMC_3-3 | 3311180 | 10.3 ± 30.9 | 64.3% | 55.3% | CWM | Control | 8.9% | 93.0% |
| CWMC_3-4 | 1625404 | 9.2 ± 20.4 | 66.6% | 56.7% | CWM | Control | 9.9% | 94.9% |
| CWMC_3-5 | 4669058 | 10.7 ± 33.5 | 86.6% | 45.4% | CWM | Control | 41.2% | 86.4% |
| CWMC_3-6 | 3196384 | 10.3 ± 31.1 | 64.1% | 55.3% | CWM | Control | 8.8% | 93.1% |
| CWMC_3-7 | 3683670 | 10.4 ± 29.0 | 87.8% | 45.7% | CWM | Control | 42.1% | 87.4% |
| CWMC_3-8 | 1518232 | 9.1 ± 16.8 | 86.6% | 45.9% | CWM | Control | 40.8% | 92.0% |
| CWMC_3-9 | 1017054 | 8.2 ± 13.9 | 84.4% | 44.4% | CWM | Control | 40.0% | 93.2% |
| CWMM_1-1 | 4801102 | 10.6 ± 34.2 | 89.4% | 46.9% | CWM | Mine | 42.6% | 87.3% |
| CWMM_1-10 | 2415236 | 10.0 ± 24.6 | 89.1% | 46.8% | CWM | Mine | 42.3% | 90.6% |
| CWMM_1-2 | 2839820 | 9.9 ± 25.9 | 87.0% | 47.3% | CWM | Mine | 39.7% | 89.8% |
| CWMM_1-3 | 4942480 | 10.8 ± 37.4 | 87.6% | 45.9% | CWM | Mine | 41.7% | 86.3% |
| CWMM_1-4 | 1255246 | 8.8 ± 16.6 | 87.6% | 46.5% | CWM | Mine | 41.1% | 92.6% |
| CWMM_1-5 | 1487352 | 9.3 ± 18.4 | 86.7% | 46.3% | CWM | Mine | 40.4% | 92.2% |
| CWMM_1-6 | 969598 | 8.4 ± 14.0 | 88.8% | 46.8% | CWM | Mine | 42.0% | 93.2% |
| CWMM_1-7 | 1510154 | 9.3 ± 18.4 | 89.4% | 46.4% | CWM | Mine | 43.0% | 92.1% |
| CWMM_1-8 | 4514418 | 10.5 ± 34.2 | 88.3% | 46.3% | CWM | Mine | 42.0% | 86.8% |
| CWMM_1-9 | 4676030 | 10.7 ± 34.8 | 87.9% | 45.7% | CWM | Mine | 42.2% | 86.9% |
| CWMM_2-1 | 1443084 | 8.8 ± 18.4 | 61.3% | 55.1% | CWM | Mine | 6.2% | 95.7% |
| CWMM_2-10 | 1078400 | 8.4 ± 16.2 | 65.6% | 56.9% | CWM | Mine | 8.7% | 96.0% |
| CWMM_2-2 | 876930 | 7.9 ± 18.6 | 61.0% | 57.6% | CWM | Mine | 3.4% | 96.9% |
| CWMM_2-3 | 4671710 | 10.4 ± 33.8 | 61.4% | 54.2% | CWM | Mine | 7.2% | 92.9% |
| CWMM_2-4 | 3989102 | 9.9 ± 24.2 | 54.7% | 30.4% | CWM | Mine | 24.2% | 90.4% |
| CWMM_2-5 | 1528502 | 9.2 ± 18.8 | 88.1% | 46.3% | CWM | Mine | 41.7% | 91.8% |
| CWMM_2-6 | 1540070 | 9.4 ± 19.6 | 88.8% | 46.8% | CWM | Mine | 42.0% | 91.8% |
| CWMM_2-7 | 1146512 | 8.7 ± 15.9 | 86.7% | 45.8% | CWM | Mine | 40.9% | 92.7% |
| CWMM_2-8 | 1260874 | 8.6 ± 17.3 | 64.2% | 55.5% | CWM | Mine | 8.7% | 95.9% |
| CWMM_2-9 | 4343052 | 10.5 ± 34.2 | 87.9% | 46.5% | CWM | Mine | 41.4% | 87.4% |
| CWMM_3-1 | 2348128 | 9.9 ± 25.3 | 88.4% | 46.1% | CWM | Mine | 42.4% | 90.4% |
| CWMM_3-2 | 3107078 | 10.3 ± 29.7 | 88.4% | 46.6% | CWM | Mine | 41.7% | 89.0% |
| CWMM_3-3 | 3175876 | 10.1 ± 29.6 | 86.2% | 45.3% | CWM | Mine | 41.0% | 89.1% |
| CWMM_3-4 | 3812658 | 10.4 ± 31.6 | 85.7% | 44.9% | CWM | Mine | 40.8% | 88.8% |
| CWMM_3-5 | 946702 | 6.7 ± 8.5 | 69.6% | 38.0% | CWM | Mine | 31.6% | 94.8% |
| CWMM_3-6 | 788384 | 7.4 ± 12.3 | 56.1% | 50.8% | CWM | Mine | 5.3% | 96.8% |
| CWMM_3-7 | 2819042 | 10.1 ± 26.3 | 86.3% | 45.8% | CWM | Mine | 40.6% | 89.8% |
| CWMM_3-8 | 1960412 | 9.3 ± 22.8 | 62.5% | 54.5% | CWM | Mine | 8.0% | 94.8% |
| CWMM_3-9 | 3266106 | 10.3 ± 31.1 | 64.8% | 54.8% | CWM | Mine | 10.0% | 93.8% |
| DGCC_1-1 | 3224134 | 10.5 ± 33.5 | 62.9% | 57.8% | DGC | Control | 5.0% | 91.6% |
| DGCC_1-2 | 3828442 | 11.0 ± 37.7 | 64.1% | 58.4% | DGC | Control | 5.7% | 90.8% |
| DGCC_1-3 | 3927684 | 11.1 ± 38.6 | 61.3% | 54.9% | DGC | Control | 6.4% | 90.8% |
| DGCC_1-7 | 3161320 | 10.4 ± 24.7 | 74.1% | 53.0% | DGC | Control | 21.1% | 90.0% |
| DGCC_1-8 | 2365628 | 10.0 ± 21.7 | 78.5% | 51.3% | DGC | Control | 27.2% | 91.0% |
| DGCC_1-9 | 1378672 | 9.2 ± 16.1 | 82.2% | 50.3% | DGC | Control | 31.9% | 93.0% |
| DGCC_2-1 | 988806 | 8.6 ± 16.8 | 62.4% | 56.9% | DGC | Control | 5.5% | 95.0% |
| DGCC_2-10 | 4169880 | 10.8 ± 28.8 | 76.8% | 47.8% | DGC | Control | 29.0% | 89.0% |
| DGCC_2-11 | 732884 | 7.9 ± 14.2 | 62.1% | 59.5% | DGC | Control | 2.7% | 96.0% |
| DGCC_2-12 | 609088 | 7.5 ± 9.5 | 81.7% | 46.6% | DGC | Control | 35.1% | 95.0% |
| DGCC_2-2 | 2910532 | 10.6 ± 34.4 | 64.3% | 58.5% | DGC | Control | 5.8% | 92.0% |
| DGCC_2-3 | 2764076 | 10.3 ± 23.2 | 77.6% | 50.6% | DGC | Control | 27.1% | 91.0% |
| DGCC_2-4 | 1586660 | 9.0 ± 21.2 | 54.2% | 55.5% | DGC | Control | -1.3% | 95.0% |
| DGCC_2-5 | 1560802 | 9.0 ± 21.2 | 60.8% | 58.5% | DGC | Control | 2.4% | 94.0% |
| DGCC_2-7 | 4436062 | 11.4 ± 41.2 | 63.5% | 58.9% | DGC | Control | 4.6% | 90.0% |
| DGCC_2-8 | 847578 | 8.3 ± 16.0 | 62.0% | 60.8% | DGC | Control | 1.2% | 96.0% |
| DGCC_2-9 | 3014542 | 10.6 ± 32.9 | 62.6% | 58.7% | DGC | Control | 3.9% | 92.0% |
| DGCC_3-1 | 1926780 | 9.7 ± 19.7 | 81.9% | 50.1% | DGC | Control | 31.8% | 92.0% |
| DGCC_3-2 | 743934 | 8.1 ± 14.7 | 63.0% | 60.3% | DGC | Control | 2.8% | 96.0% |
| DGCC_3-3 | 1423578 | 9.1 ± 16.1 | 79.3% | 51.4% | DGC | Control | 27.9% | 93.0% |
| DGCC_3-4 | 2688428 | 10.0 ± 22.2 | 75.8% | 47.4% | DGC | Control | 28.4% | 91.0% |
| DGCC_3-6 | 666722 | 7.6 ± 13.7 | 63.1% | 59.5% | DGC | Control | 3.5% | 96.0% |
| DGCC_3-7 | 4442080 | 10.8 ± 29.5 | 72.7% | 51.8% | DGC | Control | 20.9% | 89.0% |
| DGCM_1-1 | 4793280 | 11.2 ± 32.5 | 80.7% | 45.5% | DGC | Mine | 35.2% | 82.0% |
| DGCM_1-10 | 702586 | 7.9 ± 10.5 | 77.5% | 45.2% | DGC | Mine | 32.3% | 94.0% |
| DGCM_1-11 | 1303852 | 8.9 ± 15.2 | 81.5% | 46.2% | DGC | Mine | 35.4% | 91.0% |
| DGCM_1-13 | 1226950 | 9.0 ± 15.0 | 84.4% | 48.1% | DGC | Mine | 36.3% | 91.0% |
| DGCM_1-14 | 2377008 | 10.3 ± 29.5 | 65.4% | 55.8% | DGC | Mine | 9.6% | 91.0% |
| DGCM_1-2 | 2526710 | 10.2 ± 22.9 | 83.6% | 47.8% | DGC | Mine | 35.8% | 87.0% |
| DGCM_1-3 | 1298750 | 9.0 ± 19.8 | 62.1% | 52.7% | DGC | Mine | 9.3% | 94.0% |
| DGCM_1-4 | 1074746 | 8.4 ± 13.1 | 77.6% | 44.9% | DGC | Mine | 32.7% | 92.0% |
| DGCM_1-6 | 3042048 | 10.5 ± 26.0 | 84.7% | 48.0% | DGC | Mine | 36.7% | 86.0% |
| DGCM_1-7 | 2833074 | 10.5 ± 32.7 | 66.5% | 56.6% | DGC | Mine | 9.9% | 90.0% |
| DGCM_1-8 | 1715730 | 9.6 ± 24.3 | 67.1% | 57.0% | DGC | Mine | 10.1% | 92.0% |
| DGCM_1-9 | 3051964 | 10.6 ± 31.8 | 59.0% | 51.7% | DGC | Mine | 7.3% | 90.0% |
| DGCM_2-1 | 2536188 | 10.3 ± 22.9 | 83.2% | 46.9% | DGC | Mine | 36.3% | 87.0% |
| DGCM_2-2 | 3091770 | 10.8 ± 34.9 | 65.7% | 56.4% | DGC | Mine | 9.4% | 89.0% |
| DGCM_2-6 | 1453976 | 8.8 ± 16.7 | 73.9% | 42.7% | DGC | Mine | 31.2% | 91.0% |
| DGCM_2-7 | 1225942 | 9.0 ± 20.7 | 63.1% | 56.4% | DGC | Mine | 6.6% | 94.0% |
| DGCM_2-8 | 3734984 | 10.9 ± 29.7 | 82.6% | 47.3% | DGC | Mine | 35.3% | 84.0% |

Table S4. Determination of the optimal value of K for populations of L. rubellus, as calculated by Structure Harvester. The largest value of Delta K is considered to most insightful of population structure, where K=2 for all individuals, K=2 for lineage A earthworms and K=3 for those from lineage B.

| All individuals | | | | | | |
| --- | --- | --- | --- | --- | --- | --- |
| K | Reps | Mean LnP(K) | Stdev LnP(K) | Ln'(K) | Ln''(K) | Delta K |
| 1 | 10 | -1575617 | 525.3194 | NA | NA | NA |
| 2 | 10 | -1292585 | 3464.814 | 283032.6 | 247319.3 | 71.38024 |
| 3 | 10 | -1256872 | 132650.1 | 35713.34 | 25276.78 | 0.190552 |
| 4 | 10 | -1195881 | 32751.17 | 60990.12 | 49639.23 | 1.515647 |
| 5 | 10 | -1184530 | 36080.44 | 11350.89 | 165834 | 4.596228 |
| 6 | 10 | -1339014 | 315383.9 | -154483 | NA | NA |
|  | | | | | | |
| Lineage A | | | | | | |
| K | Reps | Mean LnP(K) | Stdev LnP(K) | Ln'(K) | Ln''(K) | Delta K |
| 1 | 10 | -815850 | 505.4848 | NA | NA | NA |
| 2 | 10 | -1477853 | 1035326 | -662003 | 5532606 | 5.343829 |
| 3 | 10 | -7672462 | 8367586 | -6194608 | 18194748 | 2.174432 |
| 4 | 10 | -3.2E+07 | 35276418 | -2.4E+07 | 21472241 | 0.608685 |
| 5 | 10 | -7.8E+07 | 77299182 | -4.6E+07 | 19766911 | 0.25572 |
| 6 | 10 | -1.4E+08 | 1.01E+08 | -6.6E+07 | 1.55E+08 | 1.530588 |
| 7 | 10 | -5.4E+07 | 38810439 | 89402503 | NA | NA |
|  | | | | | | |
| Lineage B | | | | | | |
| K | Reps | Mean LnP(K) | Stdev LnP(K) | Ln'(K) | Ln''(K) | Delta K |
| 1 | 10 | -418503 | 245.6799 | NA | NA | NA |
| 2 | 10 | -376789 | 9872.659 | 41714.37 | 33077.11 | 3.350375 |
| 3 | 10 | -368151 | 429.296 | 8637.26 | 3072253 | 7156.491 |
| 4 | 10 | -3431767 | 9608240 | -3063616 | 6499079 | 0.676407 |
| 5 | 10 | -1.3E+07 | 13982236 | -9562695 | 2053788 | 0.146886 |
| 6 | 10 | -2.5E+07 | 13864615 | -1.2E+07 | 27564571 | 1.988124 |
| 7 | 10 | -8662857 | 12858209 | 15948088 | NA | NA |
